# Supplementary figures and images for: Alexidine Dihydrochloride Has Broad-Spectrum Activities against Diverse Fungal Pathogens
Source: mSphere. 2018 Oct 31;3(5):e00539-18. doi: 10.1128/mSphere.00539-18 (PMC6211222; doi:10.1128/mSphere.00539-18)

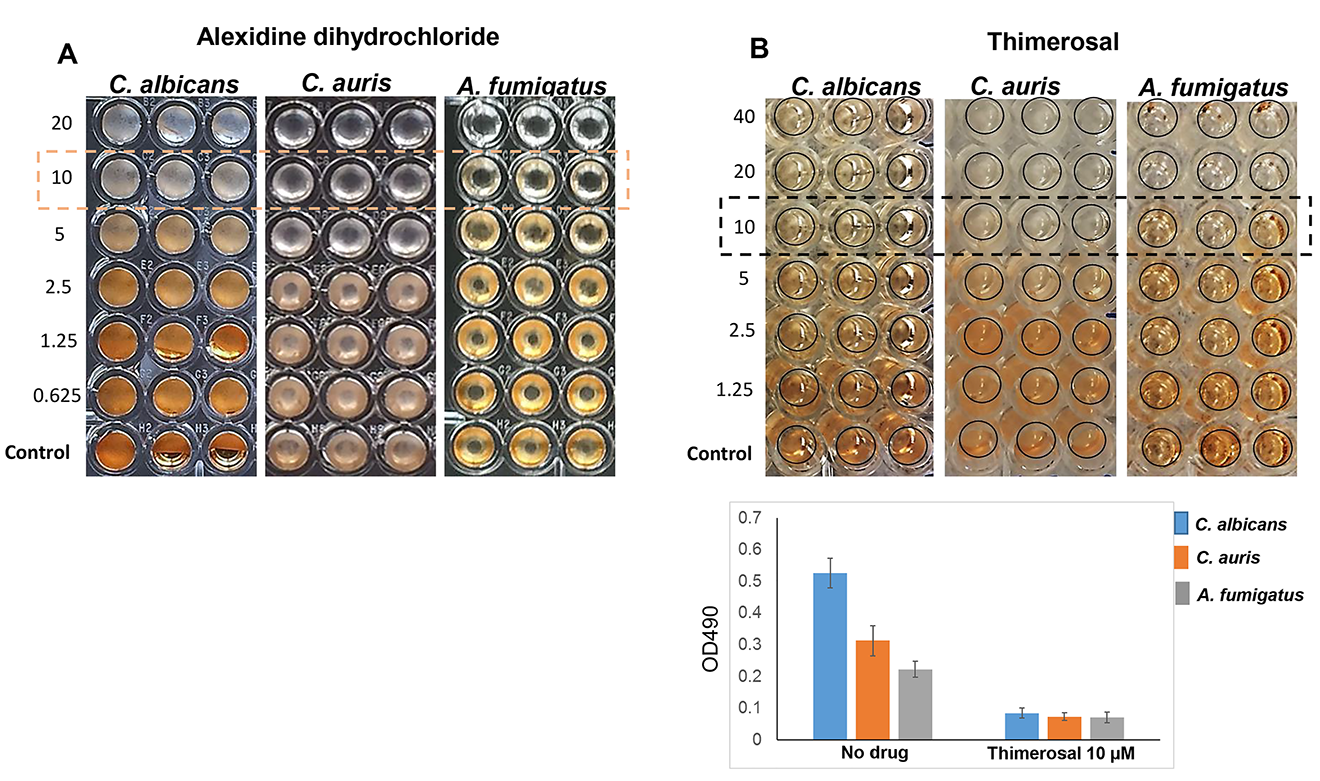

Supplement: FIG S1 [file sph006182681sf1.tif]

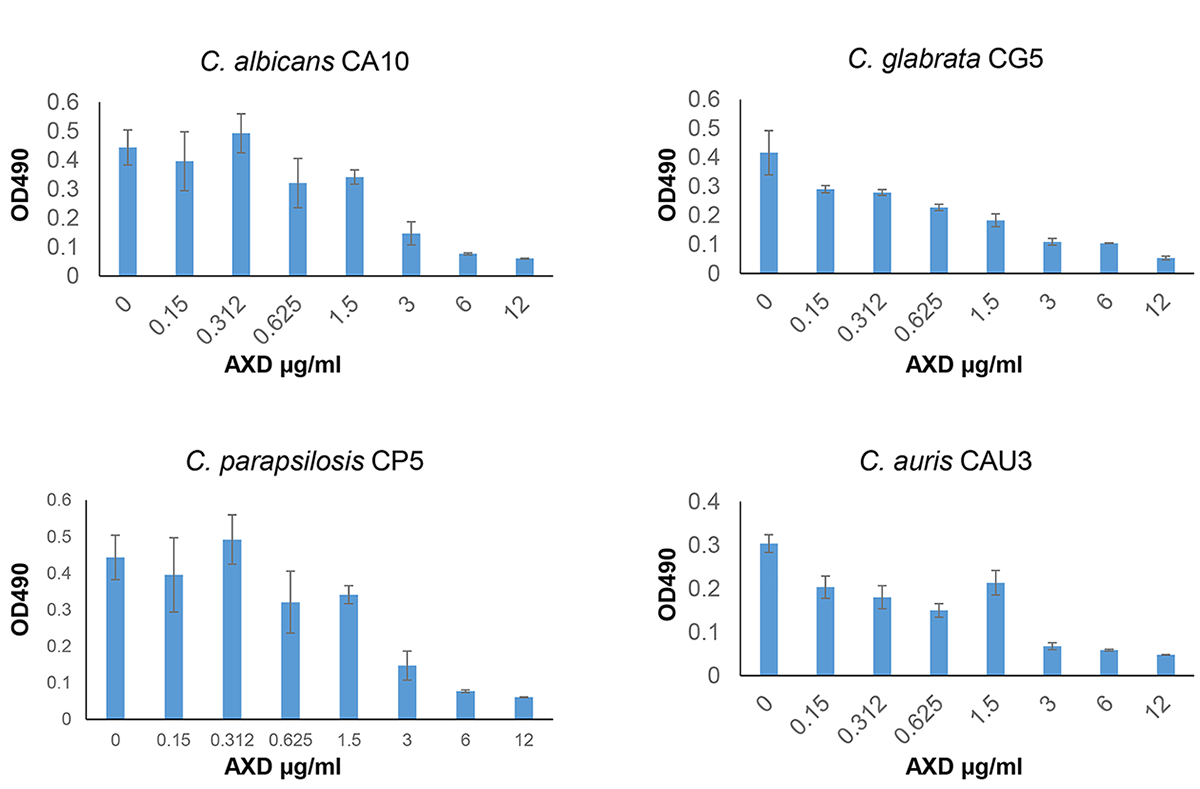

Supplement: FIG S2 [file sph006182681sf2.tif]

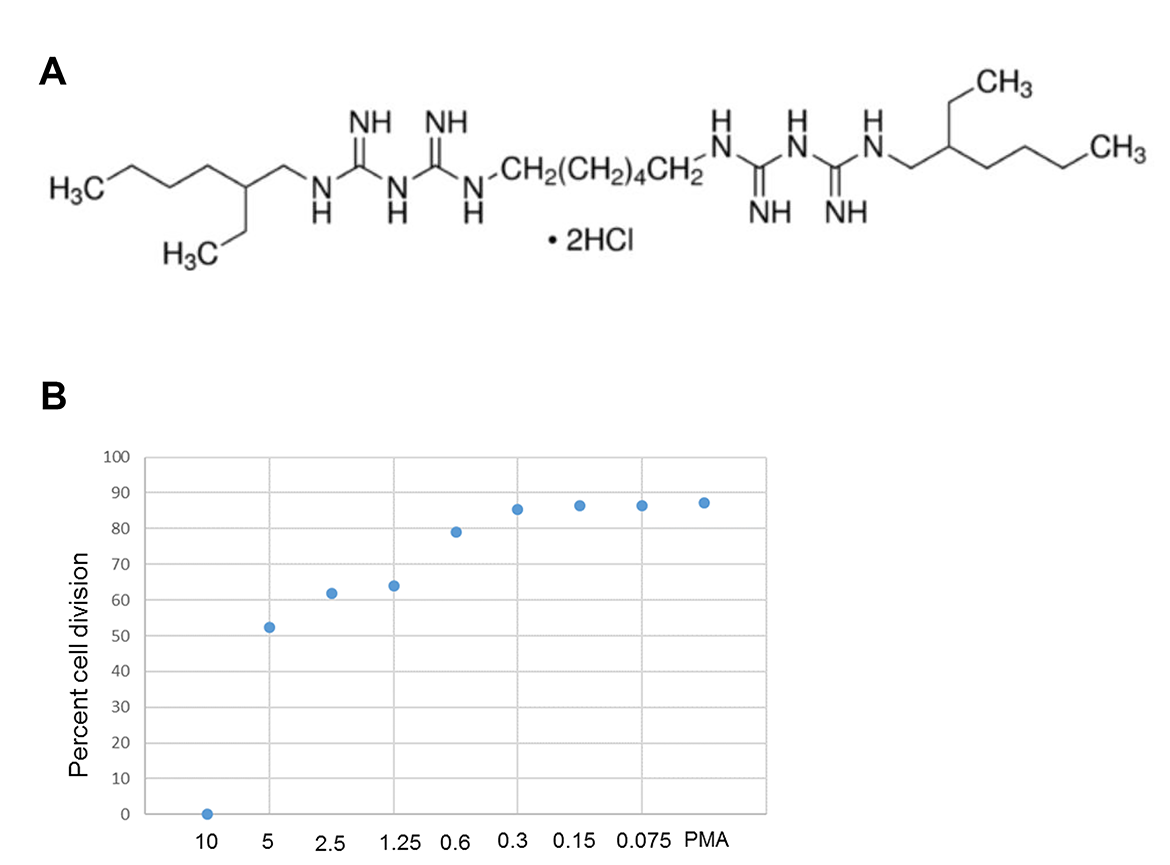

Supplement: FIG S3 [file sph006182681sf3.tif]
